# Um Tipo Incomum de Taquicardia Induzida por Marcapasso

**DOI:** 10.36660/abc.20190133

**Published:** 2020-09-11

**Authors:** Madalena Coutinho-Cruz, Guilherme Portugal, Pedro Silva-Cunha, Mário Martins-Oliveira

**Affiliations:** 1 Centro Hospitalar de Lisboa Central EPE Lisboa Portugal Centro Hospitalar de Lisboa Central EPE – Cardiologia, Lisboa - Portugal

**Keywords:** Marcapasso Artificial/efeitos adversos, Síndrome do Nó Sinusal/complicações, Taquicardia/cirurgia, Técnica de Fontan/ métodos

## Relato de Caso

**Figura 1:**
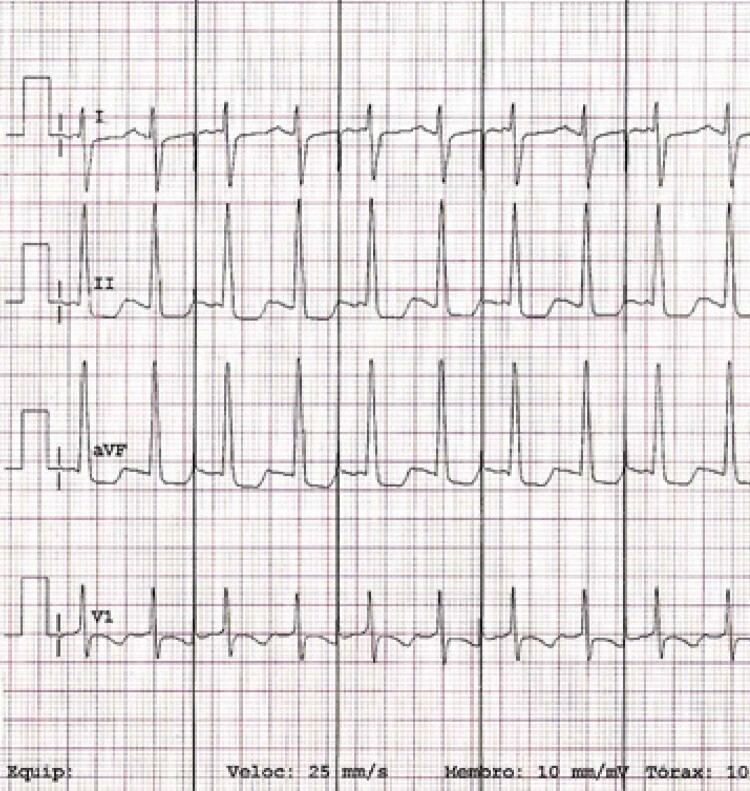
– ECG após o implante do marcapasso. Ciclo repetitivo de um batimento atrial estimulado e um batimento atrial intrínseco, ambos seguidos de um batimento ventricular intrínseco.

Uma paciente de 29 anos com ventrículo esquerdo de entrada dupla, defeito septal ventricular, má posição das grandes artérias e obstáculo subpulmonar, submetida à cirurgia de Fontan modificada, aos 9 anos de idade, apresentou síndrome de bradicardia-taquicardia com sintomas graves (palpitações e síncope). Já que não havia acesso venoso ao ventrículo direito (devido ao redirecionamento cirúrgico de fluxo sanguíneo venoso do átrio direito para a artéria pulmonar desviando dos ventrículos) e a condução AV estava normal, decidiu-se implantar um marcapasso atrial permanente. Um único eletrodo de fixação ativa foi implantado na parede lateral do átrio direito, por causa de um limiar de estimulação no apêndice atrial subotimizado. Devido a preocupações de que tanto a progressão da doença do sistema de condução quanto o uso de medicação para diminuir a frequência cardíaca pudessem levar à doença de condução AV, o que tornaria necessária a implantação de um eletrodo epicárdico mais adiante, foi utilizado um marcapasso gerador de pulso (Sorin Reply 200 DR) com *plug* na via de saída do ventrículo. No dia seguinte ao procedimento, a paciente reclamou de palpitações. A Figura 1 mostra o traçado do ECG realizado.

**Figura 2:**
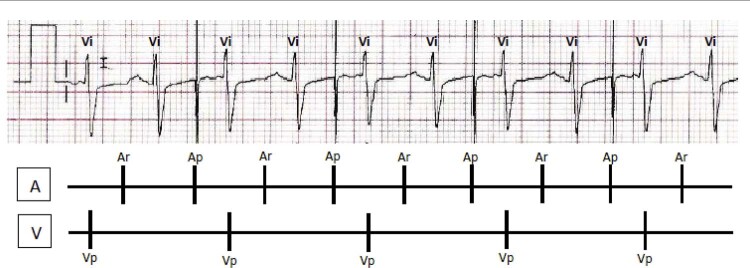
– Reprodução de eletrocardiogramas intracardíacos atrial e ventricular sobrepondo a derivação I da apresentação do ECG de superfície. A, canal atrial. Ap, evento atrial estimulado. Ar, evento atrial sentido no período refratário. V, canal ventricular. Vi, evento ventricular intrínseco. Vp, evento ventricular estimulado.

O ECG revela um ciclo repetitivo de um batimento atrial (Ap) estimulado e de um batimento atrial intrínseco (Ai), cada qual seguido de um batimento ventricular intrínseco (Vi). O intervalo Ap-Ap é de 1000 ms, o que estava em consonância com o limite mínimo da frequência programada (60 batimentos por minuto). O intervalo Ai-Ai também estava em 1000 ms e o intervalo Ap-Ai em 480 ms, o que equivale a uma frequência ventricular efetiva média de 120 bpm. O intervalo AV intrínseco é de 180 ms. Certamente, houve uma falha de sensibilidade de todos os outros batimentos atriais. Após interrogação do dispositivo, embora o limiar da estimulação e da sensibilidade estivessem adequados, observou-se que o marcapasso ainda estava com as configurações de fábrica, no modo DDD, e não no modo AAI adequado ao paciente. A Figura 2 mostra uma reprodução dos eletrocardiogramas intracardíacos sobrepondo a apresentação do ECG de superfície.

No canal atrial, um Ap é seguido de um batimento atrial intrínseco em período refratário (Ar), seguido novamente de um Ap. Como mencionado anteriormente, a via de saída ventricular no gerador estava conectada e não foi possível sentir ou estimular o ventrículo. Assim sendo, os batimentos ventriculares estimulados (Vp) são inexpressivos no que diz respeito à real ativação ventricular. Os parâmetros programados foram: limite mínimo da frequência (LRL) 60 bpm; taxa de rastreio superior 120 bpm; período refratário atrial pós-ventricular (PVARP) 280 ms; atraso AV estimulado 220 ms.

**Figura 4:**
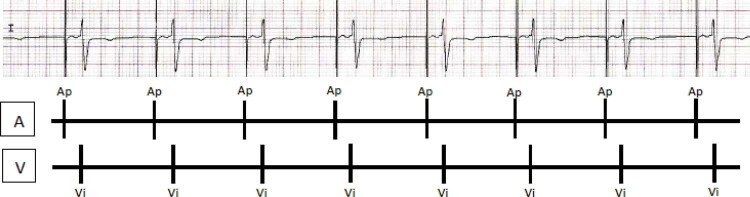
– Reprodução de eletrocardiogramas intracardíacos atrial e ventricular sobrepondo a derivação I da apresentação do ECG de superfície após a reprogramação do marcapasso. A, canal atrial. Ap, evento atrial estimulado. V, canal ventricular. Vi, evento ventricular intrínseco.

Em um marcapasso DDD, após um Ap, inicia-se um atraso AV estimulado, após o qual o marcapasso sente um Vi ou um Vp. Nesses casos, mesmo que haja um Vi através de condução nodal intrínseca, não haverá um eletrodo ventricular para sentir esse evento. O atraso AV estimulado é então seguido de um Vp que, pela mesma razão, não é representado no ECG de superfície. Após o Vp, começa o período refratário atrial pós-ventricular (PVARP). Sua principal função é evitar a sensibilidade de ondas P retrógradas, o que pode desencadear uma taquicardia mediada por marcapasso. O componente inicial do PVARP é ocupado pelo cegamento atrial pós-ventricular (PVAB), que é totalmente refratário. A partir do PVAB, o período é relativamente refratário. Durante o PVARP, os eventos atriais são sentidos e identificados como refratários (Ar) no “canal marcador” dos eventos, embora ele não modifique a sincronização dos intervalos estimulados.^1^ Desse modo, o próximo Ai não desencadeia o atraso AV e um Vp, conforme ele cai no PVARP, mas é registrado no eletrocardiograma intracardíaco como Ar. Esse Ai é seguido de um Vi através da condução nodal intrínseca, a qual, mais uma vez, não é sentida. O Ap seguinte é desencadeado após o intervalo ventrículo-atrial (VA), tendo início no último Vp, para manter o limite mínimo da frequência programada a 60 bpm (nesse caso, 780 ms). Após o marcapasso ter sido programado corretamente para o modo AAI, foi obtido o ECG apresentado na Figura 4 . Ele revela batimentos Ap a 60 bpm, cada qual seguido de um Vi sem intervenção de um Ai.

Esse tipo incomum de “taquicardia induzida por marcapasso” só é possível por causa da ocorrência simultânea de uma série de condições. Em primeiro lugar, um marcapasso com um único eletrodo atrial no modo DDD. Esse modo ocasionou um atraso AV, em seguida, um Ap e um PVARP, após o Vp. O Ai seguinte é então sentido como durante o período refratário e não reinicia os intervalos estimulados. Em segundo lugar, a ausência de um eletrodo ventricular também impede que o Vi, após o Ar, seja sentido e o intervalo VA reiniciado. Em terceiro lugar, o ritmo intrínseco do paciente durante esse período é cronometrado para ocorrer antes do final do PVARP programado, de modo que esse intervalo impede que ele seja sentido fora do período refratário. Finalmente, a condução AV intacta do paciente permite que todos os Ap e Ar sejam direcionados para o ventrículo e a frequência aumente para 120 bpm. Outro caso relatado retrata um problema semelhante, no qual um único eletrodo programado no modo DDD não reconhece um episódio de taquicardia atrial.^2^ A programação cuidadosa e um conhecimento profundo das funções da estimulação são fundamentais para o manejo desses pacientes.

**Figura 3:**
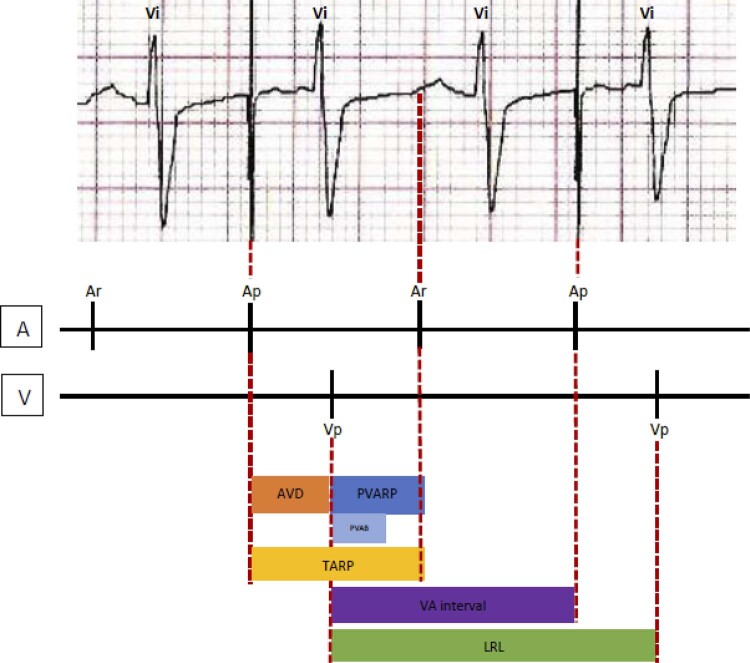
– Representação dos intervalos de estimulação do marcapasso no modo DDD. A, canal atrial. Ap, evento atrial estimulado. Ar, evento atrial sentido no período refratário. AVD, atraso AV. LRL, limite mínimo da frequência. PVAB, cegamento atrial pós-ventricular. PVARP, período refratário atrial pós-ventricular. TARP, período refratário atrial total. V, canal ventricular. Intervalo VA, intervalo ventrículo-atrial. Vi, evento ventricular intrínseco. Vp, evento ventricular estimulado.
