# Supplementary material for: Um Tipo Incomum de Taquicardia Induzida por Marcapasso
Source: Arq Bras Cardiol. 2020 Sep 11;115(1 Suppl 1):14–7. [Article in Portuguese] doi: 10.36660/abc.20190133 (PMC8386956; doi:10.36660/abc.20190133)
Supplement: Translation

# Translation: An Unusual Pacemaker-Induced Tachycardia

**Authors:** Madalena Coutinho-Cruz, Guilherme Portugal, Pedro Silva-Cunha, Mário Martins-Oliveira

**Affiliations:** 1 Portugal Centro Hospitalar de Lisboa Central EPE – Cardiologia, Lisboa - Portugal

**Keywords:** Pacemaker, Artificial/adverse effects, Sick Sinus Syndrome/complications, Tachycardia/surgery

## Case Report

A 29 year-old female patient with double inlet left ventricle, ventricle septal defect, malposition of the great arteries and subpulmonary obstacle, submitted to a modified Fontan procedure, at 9 years of age, presented with severely symptomatic brady-tachy syndrome (palpitations and syncope). Since venous access to the right ventricle was absent (due to the surgical redirection of venous blood flow from the right atrium to the pulmonary artery bypassing the ventricles) and the AV conduction was normal, it was decided to implant a permanent atrial pacemaker. A single active fixation lead was placed in the right atrial lateral wall, due to suboptimal pacing threshold in the atrial appendage. Due to concerns that either the progression of the conduction system disease or the effect of heart rate-slowing medication could lead to AV conduction disease, which would require an epicardial lead later on, a Sorin Reply 200 DR pulse generator with a plug in the ventricular port was used. The day after the procedure, the patient complained of palpitations. Figure 1 shows the ECG tracing performed.

**Figure 1:** – ECG after pacemaker implantation. Repetitive cycle of an atrial paced beat and an atrial intrinsic beat both followed by a ventricular intrinsic beat.

The ECG reveals a repetitive cycle of an atrial paced beat (Ap) and an atrial intrinsic beat (Ai) each followed by a ventricular intrinsic beat (Vi). The Ap-Ap interval is 1000 ms, which is in agreement with the programmed lower rate limit (60 beats per minute). The Ai-Ai interval is also 1000 ms and the Ap-Ai interval is 480 ms, which amounts to a mean effective ventricular rate of 120 bpm. The intrinsic AV interval is 180 ms. There was clearly an undersensing of every other atrial beat. After interrogation of the device, although pacing and sensing threshold were adequate, it was noted that the pacemaker was still in factory settings, in DDD mode instead of the patient’s appropriate AAI mode. Figure 2 shows a reproduction of the intracardiac electrograms overlaying the presenting surface ECG.

**Figure 2:** – Reproduction of the intracardiac atrial and ventricular electrograms overlaying lead I of the presenting surface ECG. A: atrial channel. Ap: atrial paced event. Ar: atrial event sensed in the refractory period. V, ventricular channel. Vi, intrinsic ventricular event. Vp, ventricular paced event.

In the atrial channel, an Ap is followed by an atrial intrinsic beat in the refractory period (Ar), followed again by an Ap. As mentioned before, the ventricular port on the generator was plugged and it was not possible neither to sense nor to pace the ventricle. As such, the ventricular paced beats (Vp) are inconsequential with respect to actual ventricular activation. Programmed parameters were: lower rate limit (LRL) 60 bpm; upper tracking rate 120 bpm; post-ventricular atrial refractory period (PVARP) 280 ms; paced AV delay 220 ms.

In a DDD pacemaker, after an Ap, a paced AV delay is started, after which there is either a sensed Vi or a Vp. In these cases, even though there is a Vi through intrinsic nodal conduction, there is no ventricular lead to sense this event. The AV delay is then followed by a Vp that, for the same reason, has no representation in the surface ECG. After the Vp, the post-ventricular atrial refractory period (PVARP) starts. Its main role is to prevent the sensing of retrograde P waves that might trigger a pacemaker mediated tachycardia. The initial component of the PVARP is occupied by the post-ventricular atrial blanking (PVAB), which is absolutely refractory. Beyond the PVAB, the period is relatively refractory. During the PVARP, atrial events are sensed and identified as refractory (Ar) on the event marker channel, though it does not modify the synchronization of the pacing intervals.^1^ Thus, the next Ai does not trigger an AV delay and a Vp, as it falls in the PVARP, but is recorded in the intracardiac electrogram as Ar. This Ai is followed by a Vi through intrinsic nodal conduction, which, again, is not sensed. The next Ap is triggered after the ventriculo-atrial (VA) interval starting at the last Vp to maintain the lower rate limit at 60 bpm (in this case, 780 ms). After correctly programming it to AAI mode, the ECG shown in Figure 4 was obtained. It reveals Ap beats at 60 bpm, each followed by a Vi with no intervening Ai.

**Figure 4:** – Reproduction of the intracardiac atrial and ventricular electrograms overlaying lead I of the surface ECG after reprogramming of the pacemaker. A, atrial channel. Ap, atrial paced event. V, ventricular channel. Vi, intrinsic ventricular event.

This unusual kind of “pacemaker-induced tachycardia” is only possible because of the simultaneous occurrence of a set of conditions. Firstly, a pacemaker with a single atrial lead was programmed in a DDD mode. This mode initiated an AV delay after Ap and a PVARP after the Vp. The next Ai is then sensed during the refractory period and does not reset the pacing intervals. Secondly, the absence of a ventricular lead also precludes the Vi after the Ar to be sensed and reset the VA interval. Thirdly, the patient’s intrinsic rhythm during this period is timed to occur before the end of the programed PVARP, so that this interval prevents it from being sensed outside the refractory period. Lastly, the patient’s intact AV conduction allows for all Ap and Ar to be conducted to the ventricle and the rate to rise to 120 bpm. Another reported case depicts a similar problem, in which a single atrial lead programmed in DDD mode fails to recognize an episode of atrial tachycardia.^2^Careful programming and deep understanding of pacing functions are crucial for the management of these patients.

**Figure 3:** – Representation of the pacing intervals of the pacemaker in DDD mode. A, atrial channel. Ap, atrial paced event. Ar, atrial event sensed in the refractory period. AVD, AV delay. LRL, lower rate limit. PVAB, post-ventricular atrial blanking. PVARP, post-ventricular atrial refractory period. TARP, total atrial refractory period. V, ventricular channel. VA interval, ventriculo-atrial interval. Vi, intrinsic ventricular event. Vp, ventricular paced event.
